# The Clinical Effect of Steroid Therapy on Preserving Residual Hearing after Cochlear Implantation with the Advanced Bionics HiRes Ultra 3D Cochlear Implant System

**DOI:** 10.3390/life12040486

**Published:** 2022-03-27

**Authors:** Magdalena Beata Skarzynska, Aleksandra Kolodziejak, Elżbieta Gos, Piotr Henryk Skarzynski, Artur Lorens, Adam Walkowiak

**Affiliations:** 1 Institute of Sensory Organs, Nadarzyn, 05-830 Warsaw, Poland; p.skarzynski@csim.pl; 2 Center of Hearing and Speech Medincus, Kajetany, 05-830 Warsaw, Poland; 3 Department of Teleaudiology and Screening, World Hearing Center, Institute of Physiology and Pathology of Hearing, Kajetany, 05-830 Warsaw, Poland; a.kolodziejak@ifps.org.pl (A.K.); e.gos@ifps.org.pl (E.G.); a.lorens@ifps.org.pl (A.L.); a.walkowiak@ifps.org.pl (A.W.); 4 Heart Failure and Cardiac Rehabilitation Department, Faculty of Medicine, Medical University of Warsaw, 03-242 Warsaw, Poland

**Keywords:** cochlear implantation, steroid administration, partial deafness treatment, hearing implants, dexamethasone, prednisone

## Abstract

(1) Background: The main aim of this study was to assess the clinical effectiveness of two different schemes of administration of steroids ((1) dexamethasone administered intravenously in comparison with (2) combination of steroid treatments: orally administered prednisone and intravenously administered dexamethasone) in comparison with a control group (no steroid administration) on hearing preservation (HP) in patients who underwent an Advanced Bionics cochlear implantation. (2) Methods: Thirty-five adult patients met the inclusion criteria. All patients were randomly divided into three subgroups depending on the scheme of steroid administration: (1) the first subgroup with only intravenously administered dexamethasone (0.1 mg per kg body weight twice a day for three days), (2) the second subgroup with a combination of methods of administration of steroids (intravenous and oral steroid therapy (dexamethasone, 0.1 mg/kg body weight twice a day plus prednisone, 1 mg/kg weight once a day for three days before surgery and after administration of dexamethasone (4th, 5th, 6th day) and after this time the dose of prednisone was reduced)) and (3) the third subgroup without steroid therapy (control group). The results were measured by pure tone audiometry (PTA) in three periods: (1) before implantation, (2) during activation of the processor (one month after implantation), and (3) 12 months after activation. Patients’ hearing thresholds before implantation were on average 82 dB HL, 77 dB HL, and 88 dB HL, respectively. (3) Results: The majority of the patients from the first subgroup had hearing preserved partially (77.8%). A similar result was observed in the second study group (oral + i.v.) (partial hearing preservation was found in 61.5% of the participants). The opposite was true in the control group; a plurality of control patients (38.5%) had no measurable hearing 12 months after the activation of the processor. (4) Conclusions: Pharmacological treatment consisting of the administration of steroids in patients who had undergone cochlear implantation with the Advanced Bionics HiRes Ultra 3D cochlear implant system may be beneficial for preserving residual hearing in patients.

## 1. Introduction

Around 466 million people globally live with disabling hearing loss resulting from a range of causes. This number is projected to rise [1]. In recent years, indications for cochlear implantation (CI) have significantly expanded. Currently, candidates for this procedure are people with partial deafness, children with congenital profound hearing loss, single-sided deafness, patients with acquired bilateral sensory hearing loss, and people over 65 [2,3,4,5]. It was believed that after the cochlear implantation surgery was performed it would be impossible to preserve the residual hearing in the patient [6,7]. Thanks to the use of new electrodes of different lengths, together with surgical techniques that ensure minimal invasiveness, the preservation of residual hearing has become possible [8]. In patients who retain their hearing, ”soft” surgery may contribute to better localization of sound and better hearing of noise [9,10]. There are more and more promising reports that describe the use of glucocorticoids in preserving residual hearing in a patient. Drugs such as dexamethasone, prednisone, and prednisolone, which are steroids with anti-inflammatory and immunosuppressive properties, have been used for these purposes [4,9,11,12].

### Aim of the Study

The aim of this study was to evaluate the clinical effectiveness of two different steroid schemes of administration and two methods of administration (intravenous or intravenous with oral administration) on the preservation of residual hearing in adult patients who underwent cochlear implantation with an Advanced Bionics cochlear system (HiRes Ultra 3D cochlear implant system with Slim J electrode) in comparison with the patients without steroid therapy. Patients who met the inclusion criteria were randomly divided into one of the three subgroups with different pharmacological treatments: (1) dexamethasone (0.1 mg per kg body weight twice a day for three days; method of administration: intravenous), (2) dexamethasone (0.1 mg per kg body weight twice a day for three days; method of administration: intravenous) and prednisone (1 mg per kg body weight once a day, method of administration: oral), (3) no steroid therapy. This research is a part of a bigger project with the purpose of optimizing the effectiveness of steroids in the preservation of residual hearing during the cochlear implantation procedure with different cochlear implantation systems (brands of cochlear implants: MED-EL Medical Electronics^®^, Advanced Bionics^®^ and Oticon Medical^®^). 

The primary endpoint of the study was to assess the average hearing thresholds (pure tone audiometry results) after implantation in comparison with the preoperative period. The assumption was that the results after surgery will be higher than before surgery. The average hearing threshold was measured at 11 frequencies (between 0.125 and 8 kHz, octaves and half-octaves). The secondary endpoint was to calculate the HP rate (hearing preservation rate) by comparing the hearing thresholds in the two different periods: (1) preoperatively and (2) postoperatively.

## 2. Materials and Methods

### 2.1. Ethical Approval and Pantients Studied 

**Figure 1:**
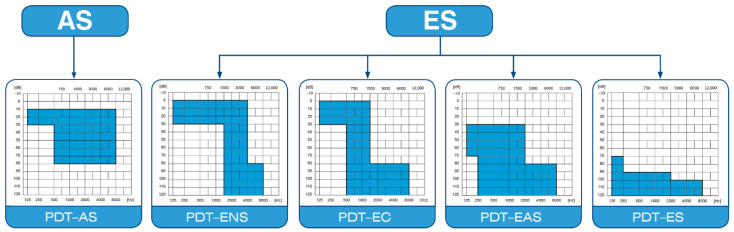
Partial deafness treatment groups for cochlear implantation. AS—acoustic stimulation; ENS—electro–natural stimulation; EC—electrical complement; EAS—electrical–acoustic stimulation; ES—electrical stimulation.

Consent has been given (number: IFPS: KB/06/2016) by the Bioethics Committee of the Institute of Physiology and Pathology of Hearing. The inclusion criteria of this study consisted of: (1) age above 18 years, (2) qualification for cochlear implantation and severe or profound hearing loss (classification of patients was conducted according to Skarzyński’s classification of partial deafness treatment (PDT): (1) PDT EAS or (2) PDT ES (Figure 1)), (3) hearing loss (between 65 and 120 dB, frequencies 0.25–1 kHz and between 75 dB and 120 dB at 2–8 kHz) according to the international HEARRING Group [4,13,14]. The exclusion criteria were as follows: (1) age below 18 years, (2) comorbidity (e.g., hypertension, cancer and/or diabetes as a contraindication for steroid therapy), (3) taking drugs that interact with steroids (e.g., immunosuppressive drugs, antidepressants).

### 2.2. The Schemes of Steroid Therapy—Two Different Algorithms and the Characteristics of Each Group

The 35 patients who were enrolled to the study were randomly divided into three subgroups. The first group consisted of 9 patients (4 women and 5 men). The age range in the first subgroup was between 34 and 71 years old (51.3 ± 11.9). The second subgroup of 13 patients (9 women and 4 men) were aged between 28 and 84 (61.4 ± 13.4). The third subgroup consisted of 13 patients (8 women and 5 men), with the age range being between 20 and 76 (59.6 ± 14.7) (Table 1). Table 1 reports their genders, ages, operated ears, and hearing thresholds in the non-operated ears. There were no statistically significant differences in any of the characteristics. 

**Figure 2:**
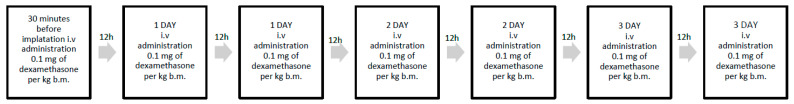
Study design for the first group, which was treated with intravenous steroid only. i.v.—inravenous; b.m.—body mass.

**Figure 3:**
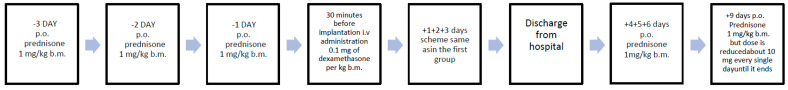
Study design for second group, which was treated with combined oral and intravenous (i.v.) steroid therapy (prolonged therapy). p.o.—per os; i.v.—intravenous; b.m.—body mass.

Each subgroup underwent a different steroid scheme of administration. In the first subgroup, dexamethasone was administered intravenously (0.1 mg per kg of body weight) 30 min before the cochlear implant surgery and for three consecutive days every 12 h (Figure 2). In the second subgroup, prednisone was administered orally (1 mg per kg of body weight) three days prior to surgery. Then, 30 min before implantation surgery, dexamethasone at a dose of 0.1 mg per kg body weight was administered intravenously (i.v.). For the subsequent three days, prednisone was administrated orally (1 mg of prednisone per kg body weight). After this time, the dose was reduced by 10 mg per day (Figure 3).

### 2.3. Measures 

The methods of this study follow those described in Skarzynska et al. (2018) and Skarżyńska et al. (2021), which also form part of our project on steroid therapy in patients undergoing cochlear implantation. The primary endpoint was measured as an average hearing threshold across all 11 frequencies (0.125–8 kHz) using both octaves and half-octaves according to the International Organization for Standardization ISO 8253-1:2010. All measurements were conducted in the same soundproof cabin by an experienced technician using the same diagnostic audiometer, the Madsen Itera II (GN Otometrics, Ballerup, Denmark) with calibrated earphones (TDH-39P) (Telephonics, New York, NY, USA). The secondary outcome measure was hearing preservation (HP), which was calculated by comparing hearing thresholds 1-year post-operation with the preoperative hearing thresholds according to the HP formula [15].

HP = (1 − ((PTA_post_ − PTA_pre_) : (PTA_max_ − PTA_pre_))) ∗ 100 [%] (1)

Equation (1). Hearing Preservation (HP) equation. (HP—hearing preservation, PTApre is pure tone average measured preoperatively, PTApost is pure tone average measured postoperatively, and PTAmax is the maximal sound intensity generated by a standard audiometer—usually 120 dB hearing level (hl)) [15].

Then, the results were divided into minimal hearing preservation (minimal HP) 0–25%; partial hearing preservation (partial HP) 26–75%; and complete hearing preservation (complete HP) > 75%. 

### 2.4. Statistical Analysis 

The assumption of normality was checked with the Shapiro–Wilk test. This assumption was not fully met, and groups were unequally sized, so non-parametric tests were used for further analysis. The Kruskal–Wallis test was performed to compare hearing thresholds from the three groups in the preoperative period and at the 12-month follow-up separately. Differences between hearing thresholds in the preoperative and 12-months post-operation were assessed separately in each group through the Wilcoxon signed-rank test. The chi-square test was used to assess the differences between the three groups in terms of hearing preservation. The analysis was conducted using IBM SPSS Statistics (v. 24).

## 3. Results

Hearing thresholds, as a primary outcome, were averaged across all frequencies (0.125–8 kHz). Preoperative outcomes were compared with those obtained 12 months after CI activation. Hearing thresholds at CI activation are shown additionally. 

Averaged hearing thresholds for all patients according to treatment type are shown in Table 2.

Hearing thresholds before implantation in the patients in all three groups were similar (H = 5.60; *p* = 0.061). In the activation period, hearing thresholds differed significantly (H = 7.86; *p* = 0.002). A difference was found between the oral + i.v. and the control group (U = 29.50; *p* = 0.005). 

Twelve months after activation, the outcomes again differed significantly (H = 6.53; *p* = 0.038). Additional comparisons revealed slight differences between the intravenous and the control group (U = 24.0; *p* = 0.020) and between the oral + i.v. and the control group (U = 46.0; *p* = 0.047). 

**Figure 4:**
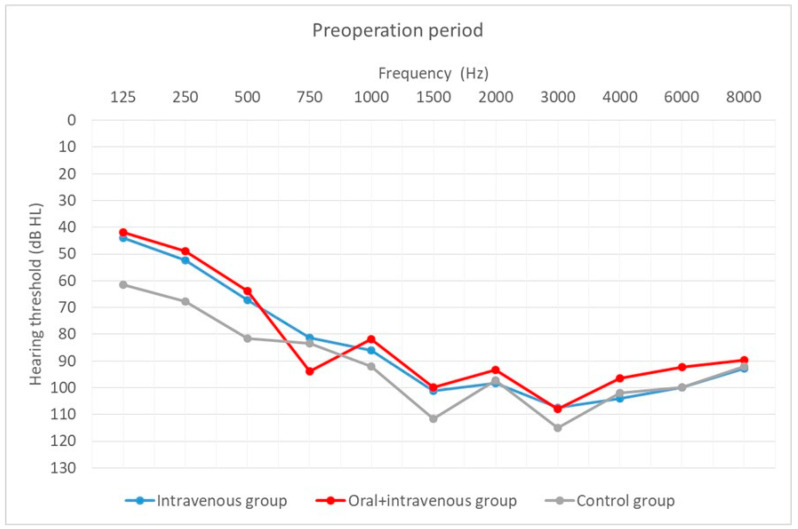
Mean hearing thresholds in the preoperative period according to treatment regime.

**Figure 5:**
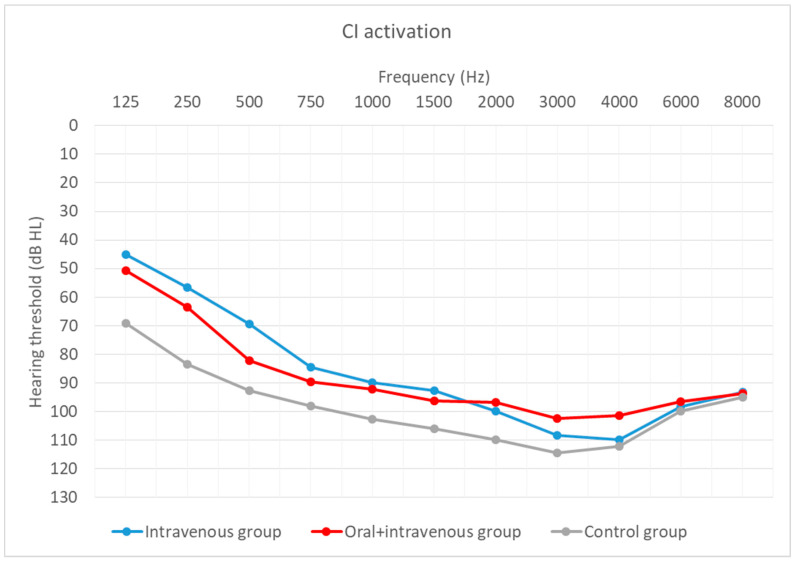
Mean hearing thresholds at CI activation according to treatment regime.

**Figure 6:**
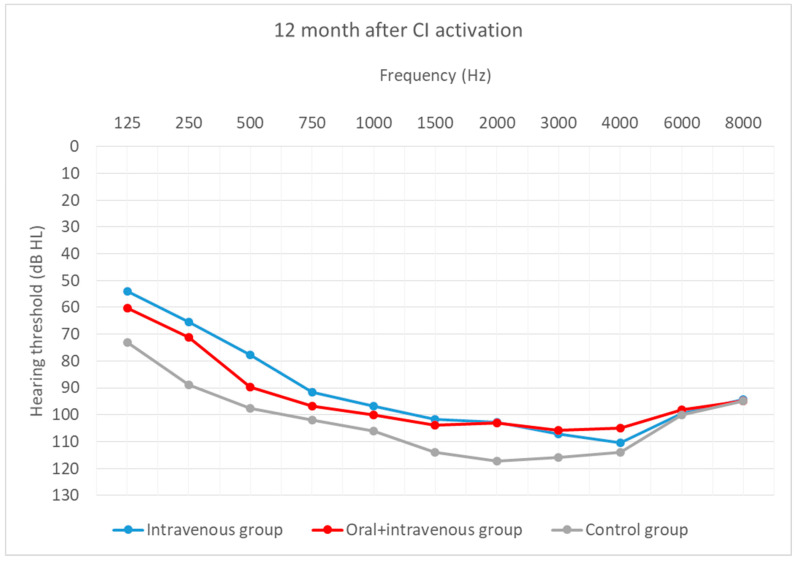
Mean hearing thresholds 12 months after CI activation according to treatment regime.

Figure 4, Figure 5 and Figure 6 depict the mean hearing thresholds across all 11 frequencies (0.125–8 kHz) in the preoperative period, at CI activation, and 12 months after CI activation.

Hearing thresholds pre-operation and 12 months after CI activation within each group were compared. In the intravenous group, the mean result (*M* = 81.74; *SD* = 10.63) worsened by about 9 dB (*M* = 91.06; *SD* = 9.92) between the pre-operative and post-operative periods, with the difference being statistically significant (*T* = 2.67; *p* = 0.008). In the oral + i.v. group, the mean difference between pre-op (*M* = 77.11; *SD* = 11.55) and 12-month outcomes (*M* = 93.53; *SD* = 10.73) was about 16 dB, and again the difference was statistically significant (*T* = 3.18; *p* = 0.001). In the control group, the deterioration was about 14 dB (from *M* = 87.73; *SD* = 9.45 to *M* = 102.13; *SD* = 8.46) and was statistically significant (*T* = 3.18; *p* = 0.001).

Hearing thresholds were also observed in patients’ non-operated ears. Generally, the hearing thresholds were stable over 12 months in the non-operated ears. In the intravenous group, the preoperative outcomes in the non-operated ears (*M* = 71,15; *SD* = 25.64) were similar to the 12-month outcomes (*M* = 70.76; *SD* = 25.32) (*T* = 0.36; *p* = 0.722). In the oral + i.v. group, the mean pre-op hearing threshold in the non-operated ears was *M* = 63.30 (*SD* = 24.35), the 12-month hearing threshold was *M* = 65.24 (*SD* = 24.46), and again the change was not statistically significant (*T* = 1.57; *p* = 0.116). In the control group, there was a slight (but statistically significant) deterioration from pre-op *M* = 64.48 (*SD* = 9.45) to 12-month post-op *M* = 66.40 (*SD* = 24.42) score (*T* = 1.99; *p* = 0.046). The mean change in the non-operated ears was 1.93 dB HL.

**Figure 7:**
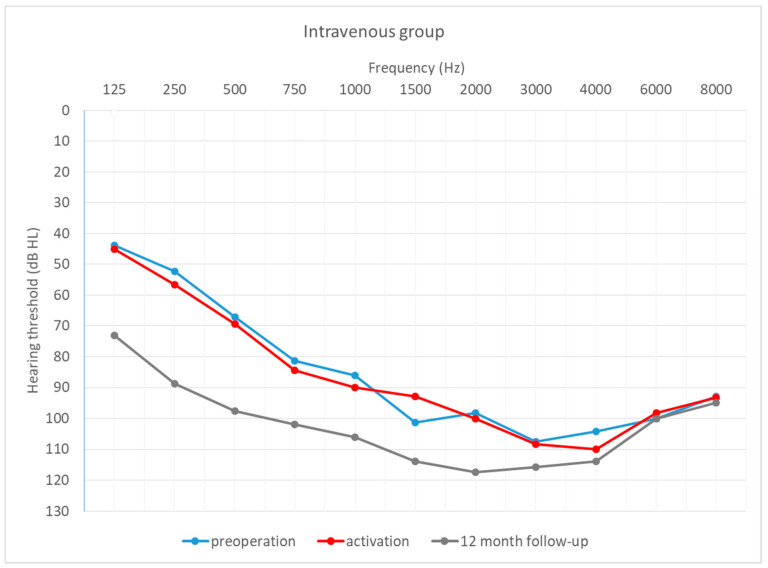
Mean hearing thresholds in the patients with standard steroid therapy (i.v. group).

**Figure 8:**
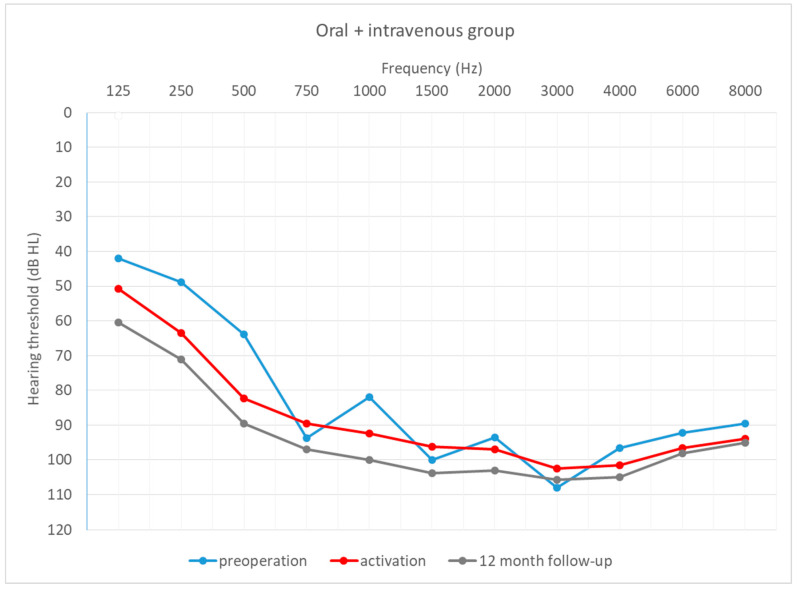
Mean hearing thresholds in the patients with prolonged standard steroid therapy (oral + i.v. group).

**Figure 9:**
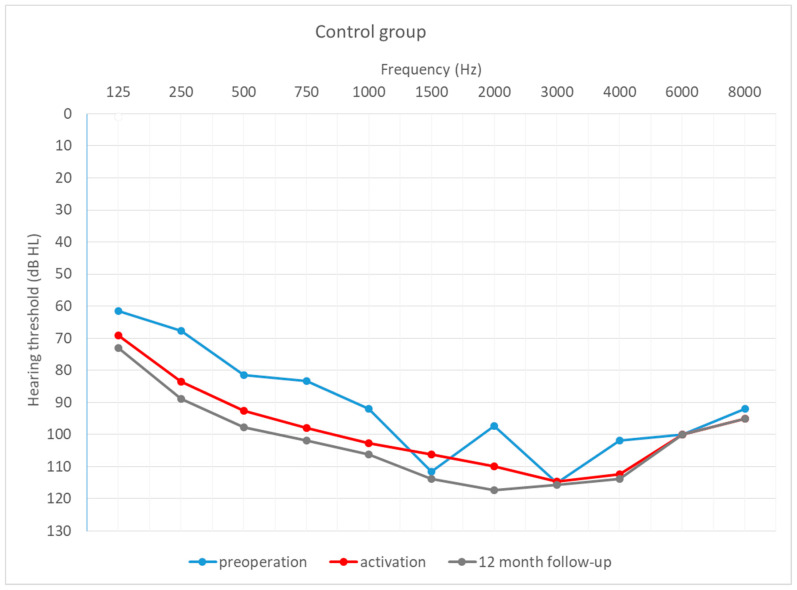
Mean hearing thresholds in the control patients.

Figure 7, Figure 8 and Figure 9 depict mean hearing thresholds across all 11 frequencies (0.125 to 8 kHz) in each group separately and in all periods.

### Hearing Preservation—The Secondary Outcome (Endpoint)

Data concerning hearing preservation converted to categories (no measurable hearing, minimal, partial, complete) are shown in Table 3.

The majority of the patients in the intravenous group had hearing preserved partially (77.8%). A similar result was observed in the oral + i.v. group (partial hearing preservation was found in 61.5% of the participants). The opposite was true in the control group. A plurality of control patients (38.5%) had no measurable hearing 12 months after activation. However, the difference between the three groups was not statistically significant (χ^2^ = 9.60; *p* = 0.143).

## 4. Discussion

According to the clinical data and publications on the subject of hearing preservation, the final clinical effect after cochlear implantation plays an important role in patients’ later life. The general conclusion in the literature is that the evidence suggests that on the one hand it is possible and on the other it is beneficial for the final hearing ability of the patient to preserve residual hearing by (1) electric stimulation and (2) pharmacotherapy (in the perioperative period). The aim of developing better atraumatic implant electrodes and improving the surgical technique is very important but depends on many different factors such as surgery skills, type of electrode in the cochlear implant, the patient’s preoperative condition (age, grade of hearing loss), and pharmacotherapy. At the same time, there is still an urgent need for pharmacologically effective and clinically approved drugs to protect hair cells in the cochlea [9,16]. As far as pharmacokinetic aspects are concerned, the most challenging route of drug administration to the inner ear is administration to the cochlear directly. To date, there is no approved drug for intracochlear delivery by the Food and Drugs Administration (FDA) or the European Medical Agency (EMA). The administration of corticoids via the routes used in this study with an Advanced Bionics cochlear implant system is in accordance with the decision of the Bioethical Committee and the summary of the product characteristics. The clinical role of pharmacotherapy (steroids) in the preservation of hearing before, during, and after cochlear implantation has been examined and assessed in many studies and animal models. According to the results of these studies, prolonged steroid administration and/or an electrode coated with steroids (e.g., dexamethasone) is beneficial to hearing and leads to a significant improvement in hearing. The results of a study from 2017 demonstrated that when an electrode is covered with a steroid (dexamethasone), it is released continuously in the cochlea [17]. When the steroid was administered intratympanically preoperatively, the results were similar and demonstrated improved hearing preservation. A comparison of the efficacy of using steroids was also assessed by Cho et al., who compared the clinical effectiveness of the preoperative (dexamethasone 5 mg/mL) and during-surgery (dexamethasone 5 mg/mL) administration of steroids for hearing protection. The results were statistically significant, but the authors did not show prolonged results. The conclusion of this study supports the beneficial clinical impact of steroid pharmacotherapy [18].

As far as the results of this study are concerned, the hearing thresholds before implantation in the patients in the three groups were similar. In the activation period, hearing the thresholds differed significantly. A difference was found between the oral + i.v. and the control group. Twelve months after activation, the outcomes again differed. Additional comparisons revealed slight differences between the intravenous and the control group and between the oral + i.v. and the control group. Hearing thresholds pre-operation and 12 months after CI activation within each group were compared. In the intravenous group, the mean preoperative outcomes worsened by about 9 dB to *M* = 91.06 (*SD* = 9.92), which was statistically significant. In the oral + i.v. group (the second subgroup), the mean difference between pre-op and 12-month outcomes was about 16 dB, and again the difference was statistically significant. In the control group, the deterioration was also about 14 dB and was statistically significant. 

The secondary endpoint in this study was hearing preservation. The majority of the patients in the intravenous group had hearing preserved partially (77.8%). Similar results were observed in the oral + i.v. group (partial hearing preservation was found in 61.5% of participants). The opposite was true in the control group, as a plurality (38.5%) showed no measurable hearing 12 months after activation. Thus, the subgroups with pharmacotherapy (steroids) gained better hearing preservation results than the non-steroid group (the control group). However, the difference between the three groups was not statistically significant. The explanation for the fact that there was no difference in hearing preservation between the residual hearing of the first and the second group may lay in the pharmacology and pharmacokinetics of dexamethasone and prednisone. Both substances are examples of the same pharmacological group (glucocorticoids), but the strength of their anti-inflammatory activities is different. Dexamethasone has the highest activity (0.75 mg of dexamethasone is equal to 5 mg of prednisone). Prednisone is a prodrug, and consequently it has to be metabolized into the active substance prednisolone in the liver. When a patient has liver functional problems (e.g., liver impairment), prednisone may be inefficiently converted into the active metabolite by cytochrome P450, and as a result he or she does not experience the pharmacological anti-inflammatory activity [19,20]. Secondly, the patients from the first and the second group showed better results in hearing preservation in comparison with the control group. Due to the lower anti-inflammatory activity of the prednisone in comparison with the dexamethasone, it may be that in this study a crucial role was played by the activity of dexamethasone, not the prednisone. Even though the duration of the therapy in the second group was longer in comparison with the first group, the oral route of drug administration is more challenging for efficient pharmacological activity. The risk of drug–food or drug–drug interactions may occur. The intravenous route of administration is better due to (1) the bioavailability being 100%, (2) the drug reaching the bloodstream immediately with full access to the entire body and allowing for rapid action (making this route the most efficient in life-threatening situations), (3) and the fact that drugs administered by the intravenous route bypass first pass metabolism [20].

According to the results of implanted ears in comparison with non-implanted ears, in the first and the second group (the steroids groups) in the two periods (preoperatively and 12 months post-operation) the hearing thresholds were stable. In the control group, according to the results of the non-operated ears, the change was statistically significant (*p* < 0.05), but the average change was equal to 2 dB and it was lower than the audiometer resolution. The audiometer resolution is equal to 5 dB (the step size was 5 dB). As a result, this size was on the one hand statistically significant but on the other it was not clinically important or significant. Consequently, and in general, in the non-operated ears of patients who were enrolled in this study in the preoperative period and 12 months post-operation on average changes in hearing thresholds were not observed. 

According to earlier studies, while the regime, scheme, dose, and duration of steroid administration was the same in all studies, the results measured by PTA were different [9,21]. The methods used to perform the study follow those described in Skarzynska et al. (2018) and Skarżyńska et al. (2021), which are part of our project on steroid therapy in patients undergoing cochlear implantation. Firstly, the ages of the enrolled patients in both previous studies were different. In the study from 2018, the age of enrolled patients was between 43 and 52, while the age range of patients enrolled in the study from 2021 was greater (between 32 and 85). In this study, the age range was between 34 and 84, which is similar to the range of age of the patients in the study with the Oticon cochlear implant system and steroids. According to previous studies, advanced age is one of the factors that in some cases is crucial to the preservation of hearing. The results of the study from 2018 proved that a combination of steroid therapy, surgical techniques, and type of electrode play an important role in hearing preservation by stabilizing the thresholds of hearing and demonstrate the superiority of combined steroid therapy (same as in our second group, dexamethasone + prednisone) at the 12-month follow-up. A total of 80% of patients had complete hearing preservation in comparison with 20% who had partial hearing preservation [9]. The results from the study with the Oticon cochlear system were different. A total of 72% of patients had no measurable hearing 12 months post-intervention. 

## 5. Limitations

One of the limitations of this study is the number of patients included (35 (9, 13, 13)). However, at the same time, due to the limited number of patients enrolled in the study and the unequal number of patients in each subgroup, non-parametric statistical analysis was used to gain reliable results. This manuscript is part of a bigger project that aims to assess the clinical effectiveness of anti-inflammatory pharmacotherapy (steroids) in protecting residual hearing in different groups of patients and with different cochlear implant systems. As a result, the possibility of enrolling equal numbers of patients in this study was limited, but in comparison with other sites in other countries in Europe, we still managed to enroll a high number of implanted patients in this study on Advanced Bionics cochlear systems.

## 6. Conclusions

According to the literature, this study is the first to compare the effectiveness of steroid (dexamethasone and prednisone) therapy in the preoperative and postoperative period under two different pharmacotherapy schemes in patients who had undergone cochlear implantation with the Advanced Bionics cochlear implant system. The hearing preservation rate was better in the two treatment groups (77.8% and 61.5%) in comparison with the control group (38.5%). However, steroid therapy may not play the most important role in the preservation of residual hearing in patients implanted with the Advanced Bionics HiRes Ultra 3D cochlear implant system.

## Figures and Tables

**Table 1:** Characteristics of the patients.

	Intravenous (i.v.) Group (n = 9)	Oral and i.v. Group (n = 13)	Control Group (n = 13)
Age	34–71; 51.3 ± 11.9	28–84; 61.4 ± 13.4	20–76; 59.6 ± 14.7
Sex	5 M; 4 F	4 M; 9 F	5 M; 8 F
Operated ear	7 R; 2 L	10 R; 3 L	6 R; 7 L
Hearing threshold in the non-operated ear	10.7–95.9; 71.2 ± 25.6	20.0–100.9; 63.3 ± 24.3	12.1–96.4; 64.5 ± 24.2

Age and hearing thresholds are expressed in years as range; mean ± standard deviation. M, male; F, female; R, right ear; L, left ear; hearing threshold (dB HL) is the average across all frequencies.

**Table 2:** Hearing threshold levels of the patients at each of the study periods.

Group		Min	Max	M	SD	Me
Intravenous	Pre	67.50	97.73	81.74	10.63	79.67
	1 m	63.18	100.00	86.21	14.36	93.18
	12 m	74.09	102.73	91.06	9.92	94.09
Oral and intravenous	Pre	55.00	100.00	77.11	11.55	72.27
	1 m	75.45	102.73	87.26	8.34	85.45
	12 m	74.09	110.45	93.53	10.73	95.91
Control	Pre	69.29	97.14	87.73	9.45	89.09
	1 m	84.55	110.45	98.57	7.40	99.55
	12 m	87.73	110.45	102.13	8.46	103.64

Min. minimum; Max. maximum; M. mean; SD. standard deviation; Me. median.

**Table 3:** Hearing preservation 12 months after CI activation according to treatment regime.

	No Measurable Hearing	Minimal	Partial	Complete
Intravenous group (IV)	0 (0.0)	0 (0.0)	7 (77.8)	2 (22.2)
Oral and IV group	1 (7.7)	2 (15.4)	8 (61.5)	2 (15.4)
Control group	5 (38.5)	2 (15.4)	4 (30.8)	2 (15.4)

Data are given as the number of patients (percentage in parentheses).

## Data Availability

The data presented in this study are available on request from the corresponding author. The data are not publicly available due to protection of personal medical data.
